# Acoustic Manipulation of Bio-Particles at High Frequencies: An Analytical and Simulation Approach

**DOI:** 10.3390/mi8100290

**Published:** 2017-09-27

**Authors:** Mohamadmahdi Samandari, Karen Abrinia, Amir Sanati-Nezhad

**Affiliations:** 1 School of Mechanical Engineering, College of Engineering, University of Tehran, North Kargar St., Tehran 14395-515, Iran; mm_samandari@ut.ac.ir; 2 Cellular and Molecular Biomechanics Laboratory, Department of Bioengineering, Imperial College London, London SW7 2AZ, UK; 3 Center for Bioengineering Research and Education, Department of Mechanical and Manufacturing Engineering, University of Calgary, Calgary, AB T2N 1N4, Canada

**Keywords:** acoustic radiation force (ARF), standing acoustic waves (SAW), bio-particle, microfluidics

## Abstract

Manipulation of micro and nano particles in microfluidic devices with high resolution is a challenge especially in bioengineering applications where bio-particles (BPs) are separated or patterned. While acoustic forces have been used to control the position of BPs, its theoretical aspects need further investigation particularly for high-resolution manipulation where the wavelength and particle size are comparable. In this study, we used a finite element method (FEM) to amend analytical calculations of acoustic radiation force (ARF) arising from an imposed standing ultrasound field. First, an acoustic solid interaction (ASI) approach was implemented to calculate the ARF exerted on BPs and resultant deformation induced to them. The results were then used to derive a revised expression for the ARF beyond the small particle assumption. The expression was further assessed in numerical simulations of one- and multi-directional standing acoustic waves (SAWs). Furthermore, a particle tracing scheme was used to investigate the effect of actual ARF on separation and patterning applications under experimentally-relevant conditions. The results demonstrated a significant mismatch between the actual force and previous analytical predictions especially for high frequencies of manipulation. This deviation found to be not only because of the shifted ARF values but also due to the variation in force maps in multidirectional wave propagation. Findings of this work can tackle the simulation limitations for spatiotemporal control of BPs using a high resolution acoustic actuation.

## 1. Introduction

Recently, tremendous amount of investigations has been devoted to manipulation of bio-particles (BPs) such as cells, liposomes, microvesicles, viruses, etc. especially using microfluidic devices [1,2,3]. Controlling the motion and position of BPs possesses a great importance in biomedical and biotechnological applications. Separation of microvesicles that carry molecular constituents of their originating cells could provide important information, useful for biomarker discovery and clinical diagnostics [4,5]. On the other hand, viruses and circulating tumor cells, as the source of several diseases, need to be detected and purified from biofluids [6,7,8]. Furthermore, cell and spheroid assembly could provide answers for tissue engineering and regenerative medicine [9,10]. However, low concentration, varying size and close physical properties of these particles to that of surrounding fluid create challenges for their manipulation [1,2,11,12]. Implementing mechanical acoustic waves is known as one effective method attracted the interest of bioengineers for BP manipulation. BPs can be immobilized using acoustic fields as a label-free, dynamic, non-invasive and cost-effective method without any special requirement neither for surrounding medium nor BP properties [13,14,15]. Therefore, acoustic manipulation methods have been broadly used in a variety of different biological applications such as fabrication of biomimetic tissues [16,17,18], filtering and purification [4,6,19] and single cell analysis [14,15,20]. In contrast to the progress in experimental acoustic-based microfluidic approaches, the underlying theoretical aspects need further investigation especially for high frequencies where BP size and acoustic wavelength are comparable [20].

The ARF is a time-averaged force exerted on particles arising from an acoustical field because of the acoustic waves scattering from the particle boundary. Theoretical studies on the acoustophoretic motion of a particle caused by the ARF started with King’s work [21] where the force applied on a small rigid particle suspended within an ideal fluid was formulated. Yosioka and Kawasima [22] tackled the rigid-particle limiting assumption by deriving a formulation for the force acting on a compressible particle. Gorkov [23] then generalized the formulation of ARF based on the gradients of the potential function in a standing pressure field. More recently, Bruus group [24,25,26] developed formulations beyond the ideal fluid assumption, however the particle size was still considered to be small (a≪λ, in which a is particle radius and λ stands for acoustic wavelength). Therefore, the validity of these approaches is limited with respect to the applied frequency ranges.

Several studies evaluated the ARF exerted on larger particles (i.e., for a ~ λ) based on Yosioka and Kawasima’s analytical approximation [27,28]. However, the formulation needed a complex program coding for numerical particle tracing methods and therefore cannot be implemented for complicated acoustic fields. Instead, Glynne-Jones et al. [29] used a numerical scheme relying on Yosioka and Kawasima’s formulation to calculate the ARF. Nevertheless, such numerical methods have limitations for coupled particle tracing because the force is calculated in frequency domain for one oscillation period without having a chance to be updated simultaneously with particle moving position in the pressure field [30]. To address this shortcoming, finite difference time domain method could be potentially a solution [31,32], although they undergo a significant computational cost [29]. Altogether, finding a predictive analytical formulation for the ARF that can be coded in particle tracing simulation is of paramount importance for high frequency acoustic particle manipulation. Given the fact that BPs are the target for majority of experimental bioengineering applications, in this study, we extend the examination of radiation force beyond the small particle assumption for BPs which their acoustic impedance is close to the surrounding fluid. An ASI model was first used to explore the ARF value exerted on BPs and quantify the resultant deformation induced to BPs. The results were then used to derive a formulation to be directly used in particle tracing simulations for spatiotemporal control of BPs in microfluidic devices (Section 3.1). The formulation was then implemented for one- and multi-directional SAWs to evaluate its potential in particle tracing investigations (Section 3.2 and Section 3.3). Finally, the range of validity for the proposed method was investigated (Section 3.4).

## 2. Methods

### 2.1. Theory

The acoustic-based fluid stress applied on each point of the BP surface generates a force with a possible deformation and motion induced to the BP boundary. The interaction between acoustic field and BP boundary could be satisfied as Equation (1) [25].

**Figure 1:**
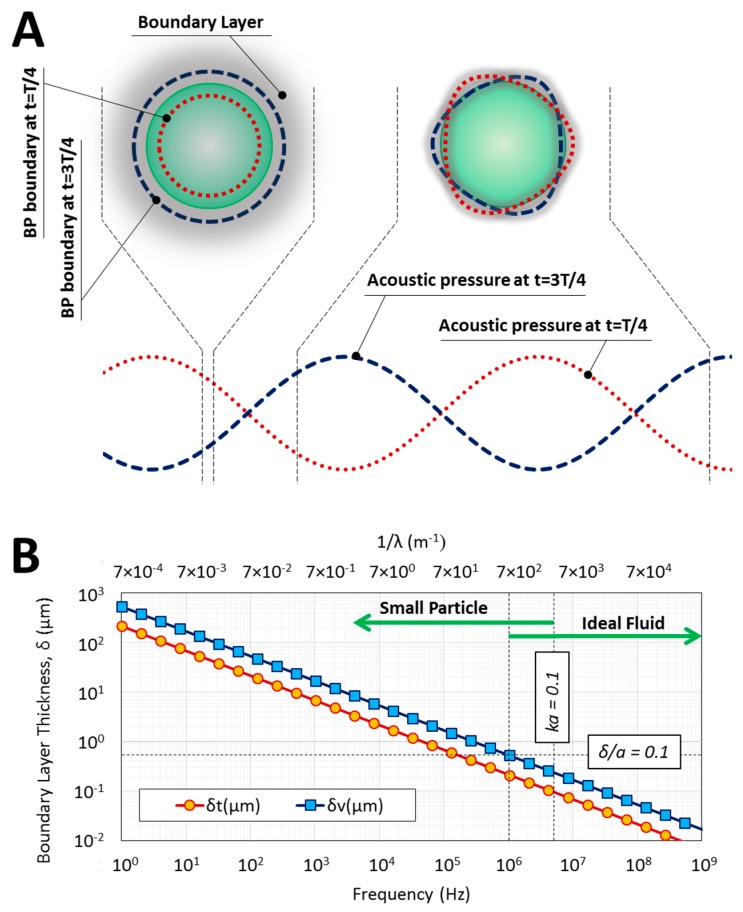
Harmonic behavior of a typical bio-particle (BP) in standing acoustic field with different applied frequencies. (**A**) Sketches of the BP under low (left) and high (right) frequency actuations; (**B**) The validity range of small particle and ideal fluid assumptions for a typical BP. In low frequencies, thermal and viscous boundary layer thicknesses ($\delta_{t}$ and $\delta_{v}$, respectively) are comparable to the BP size, therefore the fluid cannot be considered with ideal theory (see Equations (S1) and(S2)). In addition, for wavelengths much larger than particle size (i.e., ka≪1, in which k is the wave number), the BP boundary experiences either negative or positive pressure. At higher frequencies, boundary layers are thinner. On the other hand, a complex deformation and harmonic behavior are experienced by the BP given the fact that different points of the BP boundary experience different pressure values. Therefore, the BP size cannot be neglected for higher frequencies. The values are calculated based on the parameters presented in Section 2.3.

(1) P=σ·n=−np+τ·n

where P, σ and n are boundary load per unit area, fluid stress tensor and outward unit normal vector, respectively. Furthermore, p represents the pressure field and τ is the shear part of stress tensor which is non-zero only when considering a viscous fluid. To evaluate the effect of acoustic field on the overall (center of mass) movement of BPs, it is reasonable to consider time-averaged force over a full period of oscillation since it is more efficient to measure it numerically or experimentally [25]. The time-averaged (denoted by angled brackets) force, F, due to the unbalanced oscillating pressure is the ARF and therefore could be defined as Equation (2) [26,33].

(2) $$F=\langle \oint_{S\left(t\right)}\;\left(\sigma \cdot n\right)\;ds\rangle$$

where S(t) is the time dependent surface of the BP. Because of the integration complexity over a moving boundary, the force is calculated over a fixed boundary (S) enclosing the BP, meanwhile incorporating the effect of “momentum flux density” and results in the force Equation (3) [25].

(3) $$F=\langle \oint_{S}\;\left(\sigma \cdot n-\rho_{f}vv\cdot n\right)\;ds\rangle$$

where $\rho_{f}$ and v are density and velocity fields of the fluid. The instantaneous deformation and time-averaged particle motion could be calculated using Equations (1) and (3), respectively. The pressure, density and velocity fields in these equations may be determined by solving the full sets of nonlinear Navier-Stokes equations. However, to reduce the calculation cost, the perturbation theory up to the first order is used for Equation (1) as dealing with instantaneous acoustic field ($\sigma_{1}+\sigma_{2}≅\sigma_{1}$, in which the subscript numbers denote the order of the function in the perturbation method). Moreover, the perturbation theory up to second-order is used for Equation (3) since the time-average of the time harmonic first-order fields is zero [33,34]. Thus, by expanding $\sigma ,\;\rho_{f}$ and v to the second-order, the ARF is simplified to Equation (4) [25].

(4) $$F=\oint_{S}\;\left(\langle \sigma_{2}\rangle -\rho_{f0}\langle v_{1}v_{1}\rangle \right)\cdot n\;ds$$

where $\langle \sigma_{2}\rangle$ could be determined by solving the linearized compressible Navier–Stokes Equations [35]. Figure 1 shows the harmonic behavior of a BP exposed to an acoustic field, along with the validity range of the ideal fluid and small particle assumptions. The periodic pressure field in lower frequencies compress or expand the BP relative to the fluid (Figure 1A) [25]. Furthermore, the viscous and thermal boundary layers (Equations (S1) and (S2) in Supplementary Materials) are thick enough to affect the ARF (Figure 1B). Consequently, the ARF could be calculated directly from the incident first-order acoustic field for the suspended small BP within a SAW field, based on the gradients of the potential function ($U^{rad}$) as Equation (5) [25,26]

(5) $$F^{s}=-𝜵U^{rad},\;U^{rad}=V_{BP}\left(Re(f_{1})\frac{\beta_{f}}{2}\langle p_{in}^{2}\rangle -Re\left(f_{2}\right)\frac{3\rho_{f0}}{4}\langle v_{in}^{2}\rangle \right)\mathrm{for}\;\mathrm{small}\;\mathrm{particles}$$

where $V_{BP}$, $\beta_{f}$, $\rho_{f0}$, $p_{in}$ and $v_{in}$ are BP volume, isentropic compressibility and density of the fluid, and incident pressure and velocity fields, respectively. The expressions for the monopole and dipole scattering coefficients, $f_{1}$ and $f_{2}$ are given for special cases of particles in ideal [22,23], viscous [26] and thermoviscous fluids [25]. Although Equation (5) can be recovered from Equation (4) after applying the limiting assumption of small particle, there is also the possibility to use Equation (4) directly in finite element model (FEM) to obtain the ARF for an arbitrary BP size. At high frequencies of acoustic waves, the BP experiences complex harmonic behavior and introduces its size as a significant factor (Figure 1A). On the other hand, the effect of boundary layers compared to the BP size is negligible due to their infinitesimal thickness (Figure 1B). Figure S1 further demonstrates that given the close material properties of BPs and water (as fluid), the ideal fluid assumption is reliable in high frequencies of acoustic waves. As a result, the ideal fluid assumption is assumed valid in this study to evaluate the ARF exerted on BPs in high frequencies, therefore the time-averaged second order fluid stress is defined as Equation (6) [36].

(6) $$\langle \sigma_{2}\rangle =-\langle p_{2}\rangle I=-\frac{1}{2}\left(\beta_{f}\langle {p_{1}}^{2}\rangle -\rho_{f0}\langle {v_{1}}^{2}\rangle \right)I$$

Altogether, the ARF is obtained by solving the model using a linearized method to find the first order fields followed by the incorporation of Equations (4) and (6).

### 2.2. FEM Approach

To evaluate the ARF in high frequencies where the BP size and acoustic wavelength are comparable, first, an axisymmetric ASI simulation was implemented using a frequency domain solver in COMSOL Multiphysics 5. Helmholtz pressure wave in Equation (7) was solved in acoustic domain coupled with solid (BP) domain using appropriate boundary conditions (Equations (1) and (8)) on the BP membrane.

(7) 𝜵.(−𝜵p1ρf0)−ω2p1ρf0cf2=0

(8) n·(−𝜵p1ρf0)=ans

in which ω is the angular frequency. The effect of acoustic domain on solid boundary is satisfied using Equation (1) while the solid boundary affects the acoustic domain by Equation (8) obtained by setting equal the normal acceleration experienced by the fluid to that of the solid ($a_{ns})$. The second-order Equations (4) and (6) were then solved simultaneously to find the ARF. Unless otherwise mentioned, the sonication pick pressure of $p_{ac}$ = 200 kPa was applied to the BP initially located at a distance of λ/8 away from the acoustic node. Based on the results of this step, an analytical formulation was derived to examine the ARF exerted on BPs in microdevices without any need to use ASI model for particles (Equation (10); details provided in Section 3.1). To evaluate presented formulation and compare the results with previous implementation of acoustophoretic force on microparticles, simulations of BP manipulation in microdevices were conducted using “laminar flow” together with “particle tracing for fluid flow” physics interfaces (Reynolds number < 0.24).

In this work, a difference parameter (Ɗ) is defined to correlate the results of simulations based on Equation (10) and ASI accurate model, and determine the range of our formulation validity, given by Equation (9).

(9) $$Ɗ=\underset{0<D_{p}/\lambda \leq 1}{\sum }\left|F\left(D_{p}/\lambda \right)-F_{ASI}\left(D_{p}/\lambda \right)\right|/\underset{0<D_{p}/\lambda \leq 1}{\sum }\left|F_{ASI}\left(D_{p}/\lambda \right)\right|$$

where F is the ARF resulted from our target calculation and $F_{ASI}$. is the corresponding value resulted from ASI simulations in the range of *D_p_*/λ ≃ 0 to 1 (*D_p_* = 2a is the BP diameter).

### 2.3. Material Model

Selection of material properties especially bulk modulus (inverse of compressibility) needs to be performed carefully for simulating the response of BPs subjected to acoustic fields [37]. The mechanical properties used in the present work were extracted from the experimental data where the cells were subjected to identical loading conditions [38]. Detailed properties of fluid and solid used in numerical models are presented in Table 1 (in this work, subscripts f and p represent the properties of fluid and BP, respectively).

From mechanical behavior perspective, a linear elastic model (for solid) is preferred for ASI simulation of BPs [37]. Therefore, isotropic, nearly incompressible and linear elastic model was used for simulating the BP in ASI model. Additionally, the BP size was considered to be $D_{p}=10\;\mu m$.

### 2.4. Convergence and Validation

**Figure 2:**
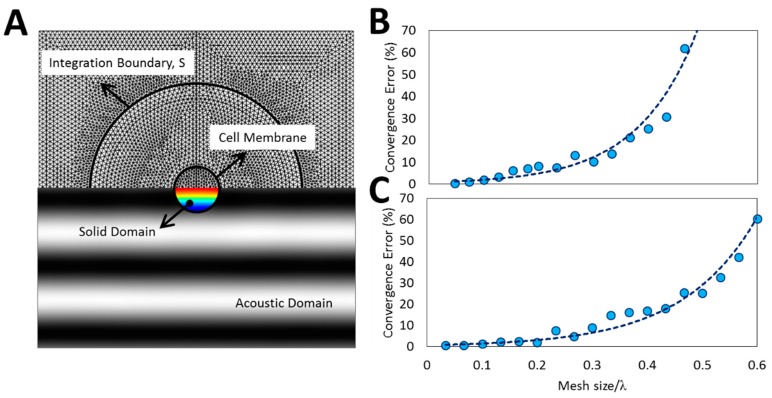
Mesh dependency analysis and convergence of finite element model (FEM) results. (**A**) Computational mesh structure for the acoustic solid interaction (ASI) model. An appropriate mesh size is selected based on the acoustic wavelength to comply with the gradients of deformation (color map inside solid domain) and acoustic pressure fields (gray-scale map of acoustic domain); (**B**,**C**) Convergence analysis of the ARF using Equation (9) for (**B**) ASI and (**C**) 2D model with proposed formulation (Equation (10)). For all simulations, a fine mesh (mesh size/λ = 1/15) was chosen to ensure the results accuracy. The graphs demonstrate that the proposed formulation has a better convergence with respect to the ASI model.

Figure 2 illustrates the ASI model along with the convergence analysis of both ASI and our proposed formulations implemented in two-dimensional (2D) simulations. The mesh size is updated dependent on the applied frequency as the gradients of pressure (in acoustic domain) and displacement (in solid domain) are inversely related to the wavelength (Figure 2A, bottom). Convergence error is plotted using Equation (9) by replacing $F_{ASI}$ with the ARF obtained using the finest mesh size. The mesh size is selected properly to ensure the convergence error stays below 1% (Figure 2B,C). The validity of the ASI model is further assessed compared to the analytical results of previous approaches (Tables S1 and S2). Subsequently, the validation of models based on Equation (10) was performed by comparing their results with the ASI model.

## 3. Results and Discussion

The deformation of a BP is analyzed through applying the scheme outlined in Section 2 to assess the ARF in a SAW field and modify previous analytical expressions. The outcomes in this section show a significant difference between the ARF resulted from our formulation and previous analytical expressions that considered small particle assumption. Therefore, it is crucial to revise previous calculations of acoustophoretic forces induced to BPs under high frequency manipulations in particle tracing approaches.

### 3.1. ARF Exerted on BPs in High Frequencies

**Figure 3:**
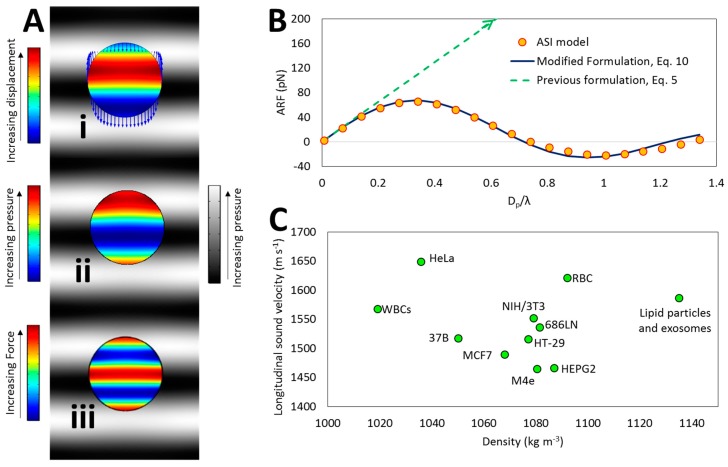
Analysis of the ARF exerted on the BP using ASI simulation. (**A**) Color map of displacement (i), internal pressure ((ii) $p_{solid}=\left(\sigma_{11}^{s}+\sigma_{22}^{s}+\sigma_{33}^{s}\right)/3$, in which $\sigma_{ii}^{s}$ is the *i*-th principle stress in the solid domain), and time-averaged force on the elements within the BP (iii). The BP is placed in an acoustic standing pressure field (gray scale) with λ = *D_p_*. In high frequencies, the elements located in various positions within the BP experience different displacements due to gradients of the pressure. Thus, the BP cannot be considered as a single point, and the resultant force over the whole BP volume needs to be calculated to obtain the ARF; (**B**) The comparison between the results of ASI model, proposed formulation (Equation (10)) in present work and previous formulations (Equation (5)). Results show a significant deviation of the ARF values in high frequencies between Equation (5) predictions and ASI results, however the proposed formulation (Equation (10)) is in a good accordance with the ASI model; (**C**) Density and longitudinal sound velocity of some BP materials [4,6,20,38,44,45]. Since deriving Equation (10) is based on the negligible difference in pressure map inside and outside the BP, the closeness of acoustic impedance of BP and surrounding fluid warrants a negligible error for the proposed formulation (see also Section 3.4).

High frequency waves need to be applied to enhance positioning resolution of acoustic-based manipulation methods for example in narrow microchannels for isolation and sorting of BPs [4] or single cell patterning [20]. Figure 3 describes the results of ASI simulation of BPs affected by standing acoustic field. Increasing the frequency promotes the gradients of the radiation potential (Equation (5)) and subsequently enhances the ARF. Furthermore, based on Equation (1), the boundary segments that are in different positions of the standing pressure field (gray scale map of Figure 3A) experience different pressures and forces. Therefore, beyond the assumption of small-particle, when the wavelength is comparable to the BP size, the instantaneous force acting on different points of the BP boundary may act in opposite directions and therefore decrease the resultant force. The displacement map of BP elements is illustrated in Figure 3A(i) where the boundary arrows indicate instantaneous normalized displacement. The elements located on various positions of BP volume experience different displacements due to the gradients of the pressure map within the BP (Figure 3A(ii)). It can be interpreted that small elements within the BP experience different (and even opposite in direction) time-averaged force based on their positions with respect to the pressure field (Figure 3A(iii)) and consequently the total time-averaged force (i.e., ARF) reduces. Therefore, for the particles with the size comparable to the acoustic wavelength, a deviation in the ARF is predicted based on the results from previous formulations (Equation (5)) with respect to our ASI simulation (Figure 3B).

Considering the assumption of small disturbance of the pressure field with presence of BP due to the minimal difference in acoustic impedance ($Z=\rho_{0}c$) of the BP and fluid, the ARF is approximated by averaging the force exerted on all small elements within the BP volume as Equation (10).

(10) $$F=\left(\int_{V_{BP}}F^{s}dv\right)/V_{BP}=-\int_{V_{BP}}𝜵\left(Re(f_{1})\frac{\beta_{f}}{2}\langle p_{in}^{2}\rangle -Re\left(f_{2}\right)\frac{3\rho_{f0}}{4}\langle v_{in}^{2}\rangle \right)dv$$

in which $F^{s}$ is the ARF field calculated using small particle assumption (Equation (5)). Equation (10) exploits the benefits of Equation (5) but here for large particles. To elaborate this further, Equation (5) calculates the ARF only based on the first-order incident acoustic field at the point that represents the particle by assuming the particle as a weak point scatterer [26]. Therefore, it could provide an ARF map without the need to the presence of the particle in the model. This fact enables the formulation, for example, to be used directly in particle tracing applications where the external fields (like pressure field) are solved and their effects (like ARF) are applied on dimensionless points as particles. Here, Equation (10) suggests that instead of calculating the ARF based on the scattering of incident wave from BP boundary (Equation (4)), it can be determined by averaging the ARF resulted from scattering of transmitted wave (through BP) from every internal element as a point scatterer. Based on the close acoustic impedance of the BP and surrounding fluid, the transmitted and incident fields are close and therefore the incident field could be used for the calculation of Equation (10). Figure 3C demonstrates that the acoustic impedance of BPs is comparable to the corresponding value for water as the surrounding fluid. Therefore, Equation (10) is applicable for most BPs (see also Section 3.4). It is noticeable that for 2D simulations (in which the pressure map repeats in 3rd dimension), $dv=2\sqrt{a^{2}-({\left(x-x_{0}\right)}^{2}+{\left(y-y_{0}\right)}^{2})}\times dA$ should be considered for force calculation in Equation (10) (where ($x_{0},y_{0}$) is the coordinate of the BP center and dA is the differential area of 2D cell). Figure 3B indicates the close correlation of Equation (10) with the results of ASI simulation.

### 3.2. One-Directional Standing Acoustic Waves

Considering a one-directional standing acoustic field as $p_{in}=p_{ac}\text{cos}\left(ky\right)$ where k is the wave number, Equation (5) simplifies to Equation (11).

(11) $$F_{1D}^{s}=4\pi \emptyset a^{3}kE_{ac}\text{sin}\left(2kh\right)$$

in which $E_{ac}=\beta_{f}p_{ac}^{2}/4$ is acoustic energy density, $\emptyset =Re(f_{1})/3+Re(f_{2})/2$ is acoustic contrast factor and h is the distance between the small particle center and acoustic node. Substituting Equation (11) into Equation (10) and performing integration, the ARF is obtained as Equation (12).

(12) $$F_{1D}=F_{1D}^{s}\times \frac{3\left[(\text{sin}\left(2\chi \right)-2\chi \;\cos\left(2\chi \right)\right]}{{\left(2\chi \right)}^{3}}=F_{1D}^{s}\times f_{m},\;\chi =ka$$

As suggested by Equation (12), when acoustic wavelength is much larger than the BP size (i.e., for χ≪1), the modifying factor ($f_{m})$ is about unity and the ARF is equal to the value predicted by Equation (5). However, increasing χ decreases $f_{m}$ until it approaches zero in Dp≃0.72λ.

**Figure 4:**
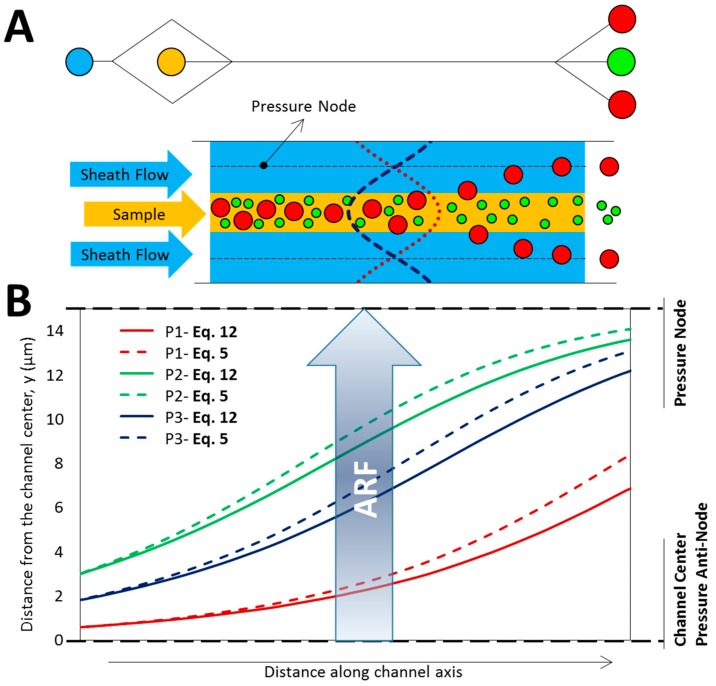
The influence of BPs size on their trajectory in a separation device. (**A**) Scheme of a typical separation microfluidic device. The ARF pushes the BPs toward acoustic nodes while exerted force is dependent on the BP size (Equation (5)). The BPs entering the sheath flow are collected from the side outlets, however smaller particles that are less affected by the acoustophoretic force, remain in the sample flow and are collected from the center outlet; (**B**) 10 µm BPs trajectory in microfluidic channel, affected by a vertical one-directional standing acoustic wave (SAW) with λ = 60 µm. Three similar BPs (P1, P2 and P3) are released from different vertical positions. Dashed lines stand for trajectories of BPs subjected to the ARF derived from conventional formulation with small particle assumption (Equation (5)). The actual trajectory considering the BP size (Equation (12)) are shown by solid lines. Since *f_m_* < 1, some BPs may not succeed to enter the sheath flow and therefore the predicted efficacy of separation reduces. The distance along the channel axis is not in scale and is based on the power and inflow channel velocity.

The primary application of acoustic-based manipulation is the separation of BPs in microdevices for upstream bioanalysis or purification of a fluid (Figure 4A) where high efficiency of the filtration is very important. Here, a one-directional acoustic field creates lines of acoustic pressure nodes/antinodes where BPs affected by the ARF aggregate based on their acoustic contrast factor. Figure 4B indicates the deviation of the BP trajectory based on the conventional formulation of ARF (dashed lines) compared to the evaluation of Equation (12). Since $f_{m}=F_{1D}/F_{1D}^{s}≃0.9$ for the acoustic field with λ=60 μm, the deviation of particle trajectory and consequently the error of separation efficiency is significant. It is noticeable that this deviation is even larger for higher frequencies as predicted by Equation (12) (Figure 3B).

### 3.3. Multidirectional Standing Acoustic Waves

**Figure 5:**
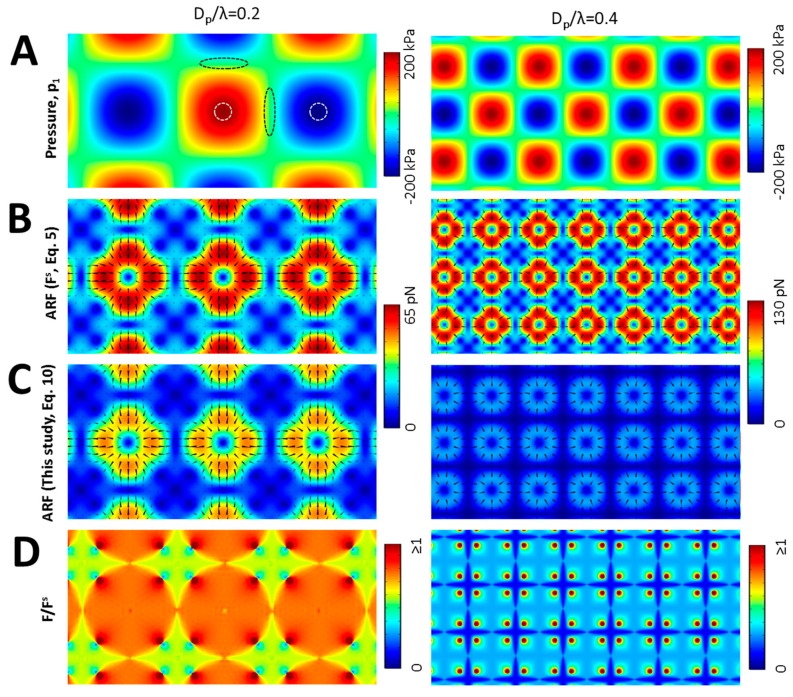
Evaluation of the ARF arising from perpendicular acoustic waves field with *D_p_*/λ = 0.2 and 0.4. (**A**) Instantaneous acoustic standing pressure map. Ellipses between positive and negative pressure areas show typical pressure nodes while circles indicate typical pressure anti-nodes; (**B**) The ARF calculated using small particle assumption. Triangles show the force direction; (**C**) The calculated ARF where the BP size is considered in equations. The results demonstrate that not only the values of actual ARF are significantly lower in comparison with previous analytical approaches but also its pattern is different particularly in higher frequencies; (**D**) The deviation of previous ARF calculations with respect to the position in standing pressure field. The variable $F/F^{s}$ implies that $F^{s}$ cannot be amended by a constant modifying factor for multidirectional acoustic fields.

SAWs could be applied in two or more directions to create a grid of pressure nodes/anti-nodes for particle patterning [18,46,47]. Increasing the frequency enhances the resolution of patterning to the scale of single cell [14,15,20]. However, successful cell patterning requires accurate calculation of the ARF as it directly affects the balance between the radiation force and interparticle forces and consequently disturbs the patterning results [20]. For a two-directional acoustic field, Figure 5 shows the deviation of the actual ARF from the prediction that considers the small particle assumption. The results show that the ARF is negligible in pressure nodes (typical ellipses in Figure 5A) or anti-nodes (typical circles) for both conventional formulation (Figure 5B) and our expression (Figure 5C). However, not only the actual values of the ARF are less than the corresponding values evaluated by previous formulations but also the ARF pattern is different. The difference in both the ARF value and its map increases with *D_p_*/λ (Figure 5D), which implies that a constant modifying factor is not appropriate for presenting a predictive formulation in multidirectional acoustic fields.

### 3.4. Range of Validity

**Figure 6:**
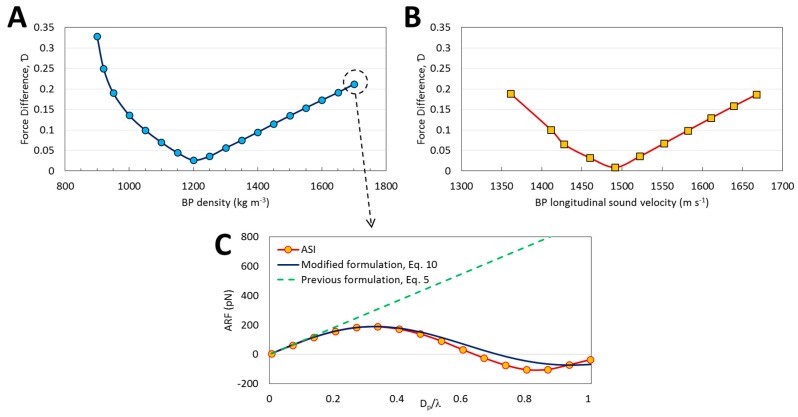
Investigation on the validity of Equation (10). The effect of (**A**) density and (**B**) longitudinal sound velocity of BPs on the force difference (Ɗ) in which the target force is the ARF calculated from Equation (10). The $c_{p}$ and $\rho_{p0}$ are kept constant based on values in Table 1, respectively for (**A**,**B**); (**C**) The ARF obtained from ASI model compared to Equation (5) and Equation (10) predictions for $\rho_{p0}≃$ 1700 kg m^−3^. Although the deviation of the BP density and longitudinal sound velocity from the optimal values shown in Figure 6A,B increases Ɗ, the predictions are still acceptable compared to the results obtained from previous formulations that use the small particle assumption (Equation (5)).

Equation (10) was derived based on the assumption of the close acoustic properties between the BPs and surrounding fluid, which allows to consider each element of the BP within an acoustic field similar to the acoustic field of the fluid domain. Thereby, the formulation can be coded and used in particle tracing in which calculations are based on one acoustic field. Figure 6 shows the validity of the assumption by evaluating the force difference resulted from the variation in BP density (while keeping $c_{p}$ constant; Figure 6A) and longitudinal sound velocity (while keeping $\rho_{p0}$ constant; Figure 6B). The longitudinal sound velocity in BP is itself dependent on the density and compressibility of the BP which are the two major material properties required for calculating acoustic contrast factor [25]. Simulations reveals that the optimum values of the BP density and sound velocity are not exactly equal to the fluid ones. To elaborate this further, transmission and reflection of the sound waves from a solid surface is not only dependent to the acoustic impedance but also to the incident and transmission angles as well as the coupling of the acoustic energy from incident waves to both longitudinal and transverse waves [48]. Here, this coupling could be affected by the material properties and system geometry. The difference between the force calculated using ASI model and Equation (10) is minimal at $\rho_{p0}≃1200$ kg m^−3^ and $c_{p}≃1500$ m s^−1^ (Figure 6A,B), while Ɗ increases with deviating from the optimum point. However, even for values other than the optimal density and longitudinal sound velocity, Equation (10) provides more accurate results than previous formulations which have been using in particle tracing analysis (Figure 6C).

## 4. Conclusions

A coupled analytical-numerical method was successfully used to evaluate the ARF exerted on BPs arising from high frequency acoustic actuation. First, a modified formulation for the ARF was derived from the results of BP deformation in an ASI model. The derived formulation was then employed to determine the difference between the ARF calculated from our proposed model and previous analytical approaches for BP separation and patterning applications in microfluidic devices. Our finding demonstrates a good accordance between our formulation and ASI model even at very high frequencies where BP size is in the order of acoustic wavelength. The proposed formulation can be used directly in particle tracing without modeling the BP itself in ASI simulations and with higher efficiency and less numerical costs. The results further indicate deviation not only in the ARF value predicted by previous analytical formulation at high frequencies but also in the ARF field map. Finally, the validity range of the proposed formulation was evaluated. The finding shows that our formulation is much more reliable than previous analytical formulations, even where a relatively large difference is indicated between the ASI results and our formulation. This implies that the results of this research can fill the gap between experimental observations and theoretical calculations in high frequencies and assist in accurate spatiotemporal control of BPs in microfluidic devices.

## Figures and Tables

**Table 1:** Detailed properties of the fluid (water at 25 °C) and BP used in the present study.

Property	Fluid ^(a)^	BP	Symbol	Unit
Density	1.0 × 10^3^	1.079 × 10^3^ ^(c)^	$\rho_{0}$	kg m^−3^
Shear modulus	-	1.67 × 10^3^ ^(c)^	*G*	Pa
Isentropic compressibility	4.433 × 10^−10^ ^(b)^	3.78 × 10^−10^ ^(c)^	*β*	Pa^−1^
Thermal expansion	2.748 × 10^−4^	2.0 × 10^−4^ ^(d)^	*α*	K^−1^
Specific heat capacity at constant pressure	4.181 × 10^3^	3.421 × 10^3^ ^(e)^	*h_c_*	J kg^−1^K^−1^
Ratio of specific heats	1.012	1.012 ^(f)^	*γ*	1
Thermal conductivity	6.095 × 10^−1^	4.9 × 10^−1^ ^(e)^	*k_t_*	Wm^−1^K^−1^
Longitudinal (compressional) wave speed	1.502 × 10^3^	1.566 × 10^3^ ^(g)^	*c*	m s^−1^
Transvers (shear) wave speed	-	1.244 × 10^3^ ^(h)^	*c_s_*	m s^−1^
Shear viscosity	8.538 × 10^−4^	-	*μ*	Pa s
Bulk viscosity	2.4 × 10^−3^	-	*μ_b_*	Pa s

^(a)^ Material properties for water from Ref. [25]; ^(b)^ Calculated as $1/\left(\rho c^{2}\right)$ from Ref. [39]; ^(c)^ Properties of NIH/3T3 cell from Ref. [38]; ^(d)^ Approximate value for tissue from Ref. [40,41]; ^(e)^ thermal properties of tissue from Ref. [42]; ^(f)^ Assumed to resemble water; ^(g)^ Calculated as $c=\sqrt{3\left(1-\upsilon /1+\upsilon \right)/\rho_{0}\beta }$ from Ref. [39], in which υ≃0.5 is Poisson ratio calculated using *G* and bulk modulus κ=1/β; ^(^^h)^ Calculated as $c_{s}=\sqrt{G/\rho_{0}}$ from Ref. [43].

## Supplementary Materials

The following are available online at www.mdpi.com/2072-666X/8/10/290/s1, Figure S1: Comparison between ideal, viscous and thermoviscous fluid in calculation of the acoustic radiation force (ARF) applied on the small bioparticle (BP) suspended in an acoustic standing field. (A) Acoustic contrast factor of the BP in water as a function of δ_v_/a. Ideal theory suggests a steady contrast factor while viscous and thermoviscous theories predict a variable contrast factor. However, the difference between these values are negligable; (B) The ARF error when using an ideal fluid compared to viscous (green dashed line) or thermoviscous (blue solid line) fluid. Results demonstrate that the maximum ARF error with an ideal fluid assumption is less that 1%, however the error significantly decreases with increasing the acoustic frequency. The detailed properties are presented in Table 1; Table S1: Comparison of the ARF values obtained from different studies along with the results of ASI model in present study; Table S2: Properties of the cell/particle and fluid (water) for calculation of ARF results shown in Table S1.
