# Supplementary material for: Acoustic Manipulation of Bio-Particles at High Frequencies: An Analytical and Simulation Approach
Source: Micromachines (Basel). 2017 Sep 27;8(10):290. doi: 10.3390/mi8100290 (PMC6190359; doi:10.3390/mi8100290)
Supplement: Supplementary file 1 [file micromachines-08-00290-s001.pdf]

micromachines

Supplementary Information: Acoustic Manipulation of Bio-Particles at High Frequencies: An Analytical and Simulation Approach

Mohamadmahdi Samandari, Karen Abrinia and Amir Sanati-Nezhad

SI. 1 Viscous and Thermoviscous Fluid

In viscous and thermoviscous theories, viscous and thermal boundary layers can affect the acoustic radiation force (ARF) exerted on biological particles (BPs). Thermal and viscous boundary layer thicknesses (δ_t and δ_v, respectively) could be obtained as Equations (S1) and (S2) (parameters are defined in Table 1) [1].

$$\delta_t = \sqrt{k_t/(\pi \rho_{f0} h_c f)} \tag{S1}$$

$$\delta_v = \sqrt{\mu_f/(\pi \rho_{f0} f)} \tag{S2}$$

in which f is the applied frequency of acoustic field. Since the BPs and surrounding fluid have similar properties, the difference between the ARF calculated based on the assumption of ideal or thermoviscous/viscous fluids are negligible. Figure S1 indicates this negligible difference based on the previous analytical expressions [1,2] and using properties described in Table 1.

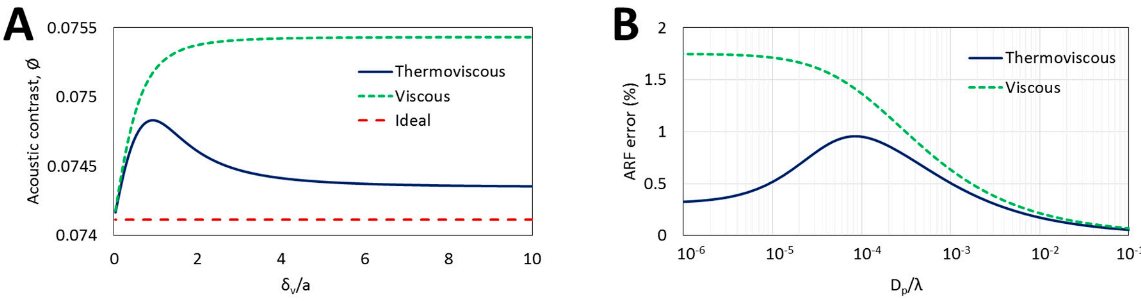

Figure S1. Comparison between ideal, viscous and thermoviscous fluid in calculation of the acoustic radiation force (ARF) applied on the small bioparticle (BP) suspended in an acoustic standing field. (**A**) Acoustic contrast factor of the BP in water as a function of δ_v/a. Ideal theory suggests a steady contrast factor while viscous and thermoviscous theories predict a variable contrast factor. However, the difference between these values are negligable; (**B**) The ARF error when using an ideal fluid compared to viscous (green dashed line) or thermoviscous (blue solid line) fluid. Results demonstrate that the maximum ARF error with an ideal fluid assumption is less that 1%, however the error significantly decreases with increasing the acoustic frequency. The detailed properties are presented in Table 1.

SI. 2 Validation

A good accordance of our method with previous studies is shown in Table S1. The ARF values calculated in this table are based on applying a 1 MHz ultrasound field with acoustic pressure of 200 kPa while the properties of the cells/particles and fluid are provided in Table S2.

Table S1. Comparison of the ARF values obtained from different studies along with the results of ASI model in present study.

Material	ARF [pN], This study	ARF [pN], Y & K [3]	ARF [pN], Bruus [2]	ARF [pN], Gorkov [2]
NIH/3T3	2.15	2.15	2.15	2.15
MCF7	7.18	7.18	7.19	7.18
RBC	0.840	0.839	0.842	0.839
Polystyrene, Styron 666	5.10	6.70	5.10	5.10
Stainless Steel, 347	21.81	21.85	23.40	21.80

Table S2. Properties of the cell/particle and fluid (water) for calculation of ARF results shown in Table S1.

Particle	Density (kg/m^3)	Bulk modulus (Gpa)	Shear Modulus (kPa)	Radius (μm)
NIH/3T3 cell line [4]	1079	2.60	1.67	5
MCF7 cell line [4]	1068	2.37	0.103	9
Red blood cell (RBC) [5]	1092	2.87	1.5	3.25
Polystyrene (PS), Styron 666 [6]	1050	4.20	1.39E6	5
Stainless Steel, 347 [6]	7890	163	75.8E6	5
**Fluid**	**Density (kg/m^3)**	**Bulk modulus (Gpa)**	**Shear Viscosity (mPa.s)**	**Speed of Sound (m/s)**
Water [7]	997	2.23	0.89	1497

References

1. Karlsen, J.T.; Bruus, H. Forces acting on a small particle in an acoustical field in a thermoviscous fluid. *Phys. Rev. E* **2015**, *92*, 043010.
2. Settnes, M.; Bruus, H. Forces acting on a small particle in an acoustical field in a viscous fluid. *Phys. Rev. E* **2012**, *85*, 016327.
3. Yosioka, K.; Kawasima, Y. Acoustic radiation pressure on a compressible sphere. *Acta Acust. United Acust.* **1955**, *5*, 167–173.
4. Hartono, D.; Liu, Y.; Tan, P.L.; Then, X.Y.S.; Yung, L.-Y.L.; Lim, K.-M. On-chip measurements of cell compressibility via acoustic radiation. *Lab Chip* **2011**, *11*, 4072–4080.
5. Collins, D.J.; Morahan, B.; Garcia-Bustos, J.; Doerig, C.; Plebanski, M.; Neild, A. Two-dimensional single-cell patterning with one cell per well driven by surface acoustic waves. *Nat. Commun.* **2015**, *6*, 8686.
6. Selfridge, A.R. Approximate material properties in isotropic materials. *IEEE Trans. Sonics Ultrason.* **1985**, *32*, 381–394.
7. Muller, P.B.; Bruus, H. Numerical study of thermoviscous effects in ultrasound-induced acoustic streaming in microchannels. *Phys. Rev. E* **2014**, *90*, 043016.
